# Leptin Receptor-Deficient *db/db* Mice Show Significant Heterogeneity in Response to High Non-heme Iron Diet

**DOI:** 10.3389/fnut.2021.741249

**Published:** 2021-09-27

**Authors:** Sabine Paeschke, Karsten Winter, Ingo Bechmann, Nora Klöting, Matthias Blüher, Petra Baum, Joanna Kosacka, Marcin Nowicki

**Affiliations:** ^1^Institute of Anatomy, University of Leipzig, Leipzig, Germany; ^2^Helmholtz Institute for Metabolic, Obesity and Vascular Research (HI-MAG) of the Helmholtz Zentrum Munchen at the University of Leipzig, Leipzig, Germany; ^3^Department of Medicine, University of Leipzig, Leipzig, Germany; ^4^Department of Neurology, University of Leipzig, Leipzig, Germany; ^5^Applied Molecular Hepatology Lab, Department of Visceral, Transplant, Thoracic and Vascular Surgery, University of Leipzig Medical Center, Leipzig, Germany

**Keywords:** iron, obesity, diabetes mellitus, metabolism, inflammation, *db/db* mice

## Abstract

Recent studies have shown an association between iron homeostasis, obesity and diabetes. In this work, we investigated the differences in the metabolic status and inflammation in liver, pancreas and visceral adipose tissue of leptin receptor-deficient *db/db* mice dependent on high iron concentration diet. 3-month-old male BKS-Leprdb/db/JOrlRj (*db/db*) mice were divided into two groups, which were fed with different diets containing high iron (29 g/kg, *n* = 57) or standard iron (0.178 g/kg; *n* = 42) concentrations for 4 months. As anticipated, standard iron-fed *db/db* mice developed obesity and diabetes. However, high iron-fed mice exhibited a wide heterogeneity. By dividing into two subgroups at the diabetes level, non-diabetic subgroup 1 (<13.5 mmol/l, *n* = 30) significantly differed from diabetic subgroup two (>13.5 mmol/l, *n* = 27). Blood glucose concentration, HbA1c value, inflammation markers interleukin six and tumor necrosis factor α and heme oxygenase one in visceral adipose tissue were reduced in subgroup one compared to subgroup two. In contrast, body weight, C-peptide, serum insulin and serum iron concentrations, pancreatic islet and signal ratio as well as cholesterol, LDL and HDL levels were enhanced in subgroup one. While these significant differences require further studies and explanation, our results might also explain the often-contradictory results of the metabolic studies with *db/db* mice.

## Introduction

Obesity is one of the major metabolic disorders today and an important risk factor for the development of type 2 diabetes (T2D) (1, 2). In 2016, more than 1.9 billion adults were overweight and among those 650 million had obesity. Noteworthy, in 2017, ~462 million individuals were affected by T2D corresponding to 6.28% of the world's population and over one million deaths per year can be attributed to diabetes alone (WHO) (1, 3). Despite recent advances in our understanding how obesity develops and contributes to metabolic disorders, the precise pathomechanisms are not entirely clear (1, 2). In particular, it remains open why only a subgroup of people with obesity and T2D develop complications. Rodent models may help to identify the root causes and pathogenic factors underlying the large human variation obesity related diseases and T2D complications. The leptin-receptor-deficient C57BLKS (BKS) inbred mouse (*db/db*) strain represents one of the first animal models of T2D (4). This strain is frequently used to study T2D mechanisms because of the many similarities to the development of human obesity-related T2D. Development of diabetes in *db/db* mice seems to be mediated by the interaction between the leptin receptor deficiency and additional genetic factors (5–9). T2D in *db/db* mice is associated with obesity and is characterized by insulin resistance as an initial alteration which subsequently leads to pancreatic ß-cell failure (10).

Recently, an association between iron homeostasis, obesity and T2D has been shown, but the link between dietary iron and metabolic dysfunction is poorly defined (11–13). Iron plays an important role in many biological functions such as oxygen binding and transport, oxygen metabolism and cell development (14). Iron is also a co-factor for heme and non-heme-dependent enzymes and it is involved in mitochondrial energy generation (14, 15). Free iron increases formation of reactive oxygen species (ROS) via Fenton reaction (16). In turn, increased ROS levels could induce degradation of intracellular proteins, lipids and DNA (17). Interestingly, iron overload in pancreatic ß-cells causes cell damage and apoptosis, leading to impaired insulin synthesis and secretion (18). Apoptosis of pancreatic ß-cells, liver dysfunction as well as peripheral insulin resistance promote the development of T2D (16–18). Notably, the uptake of heme-bound iron, but not of non-heme iron, is positively associated with an increased risk of T2D (11, 12, 19). Moreover, elevated free iron concentrations, accumulation of iron-related markers such as ferritin and transferrin and the expression of heme oxygenase 1 (Hmox1) in adipose tissue of mice and humans correspond with higher inflammatory process and lower adipogenesis (20–26). High iron burden in adipose tissue induces insulin resistance, but the mechanisms underlying adipose iron accumulation remain also unknown (13).

In our recent work, we investigated the role of iron in the development of peripheral diabetic neuropathy, one of the most common complications of T2D. We found that dietary iron deficiency, but not non-heme iron overload, leads to peripheral nerve dysfunction in obese diabetic *db/db* mice (27). In this study, the positive effect of high non-heme iron diet on nerve conduction velocities and reduced pro-inflammatory milieu have been shown in sciatic nerves of these animals (27). Generally, *db/db* mice fed with high iron diet had a lower blood glucose level and HbA1c content as well as higher serum insulin concentrations (27). Surprisingly, a detailed analysis of glucose, lipid and iron metabolic parameters revealed two subgroups of *db/db* mice fed with high non-heme iron diet. Recently, our research group has investigated the influence of dietary non-heme iron on the development of peripheral neurological complications in the metabolic syndrome [leptin-deficient *ob/ob* mice, (28)], streptozotocin (STZ)-induced type 1 [STZ-rats, (29)] and type 2 [*db/db* mice, (27)] diabetes mellitus. In these studies, the most important criterion was blood glucose level as a determinant of the diabetic status of the experimental animals. It is generally known that hyperglycemia (blood glucose concentration >13.5 mmol/l) is one of the most important factors in the development of peripheral neuropathy. In all of our studies to date, the same high iron diet was used and, with the exception of the last work with *db/db* mice, no significant differences were found in response to high dietary iron levels within one animal group (27–29). Therefore, it was surprising to observe the dual response of *db/db* diabetic mice to high iron diet (27). Notably, reports on the effect of iron on the patient diabetic status and development of the diabetic complications are contradictory (11, 13). In the present work, we aim to investigate differences in the metabolic status, as well as inflammation markers in liver and adipose tissue between two high iron diet fed *db/db* mouse subgroups. The detailed characterization of these two subpopulations of *db/db* mice reacting differently to dietary iron might help to explain the different effects of this nutrient on individual patients and to choose the appropriate adjuvant therapy against iron homeostasis disorders.

## Materials and Methods

### Animals and Phenotypical Analyses

**Figure 1:**
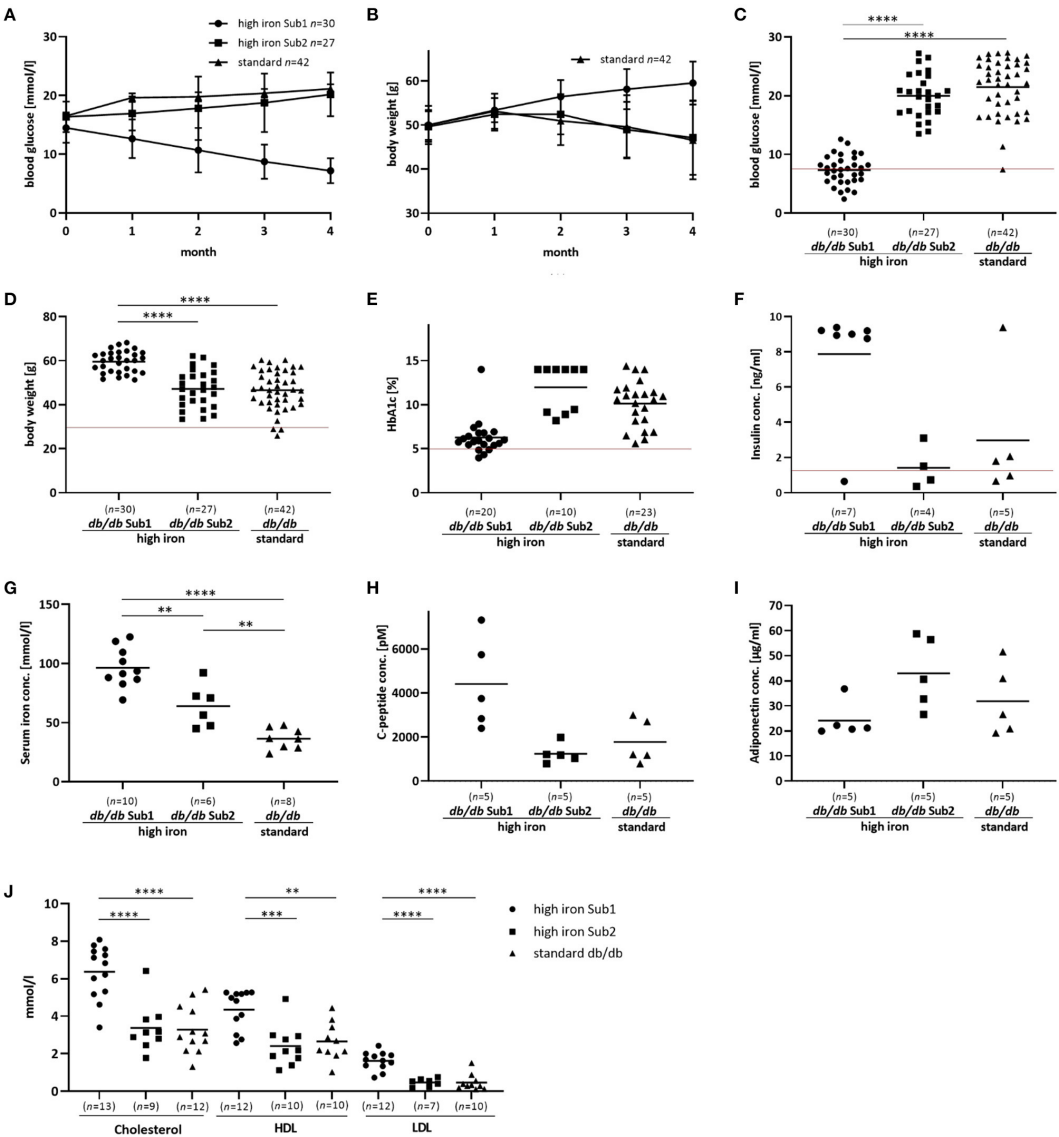
Phenotype and additional serum analyses of the investigated mouse groups. Blood glucose, body weight and serum analyses of *db/db* mice fed with high iron and standard diet for 4 months. **(A,B)**: Blood glucose concentration and body weight during 4 months' diet. Values represents means ± SD **(C)**: Blood glucose concentration in Sub1 fed with high iron diet is significantly reduced compared to Sub2 and standard group after 4 months' diet (ANOVA). **(D)**: Body weight is significantly increased in Sub1 (ANOVA). **(E)** HbA1c content at the end of experiment (Kruskal Wallis test). **(F)** Insulin concentration in mouse serum (Kruskal Wallis test). **(G)**: Serum iron concentration (ANOVA) is higher in subgroup 1 than in subgroup 2 and standard *db/db* group. **(H)**: C-peptide concentration in serum (ANOVA). **(I)**: Adiponectin serum concentration (ANOVA). **(J)**: Measurements of cholesterol, HDL and LDL (ANOVA). A significant reduction in blood glucose concentration with lower HbA1c levels is observed in subgroup 1 compared to the others. Values represent means ^**^p < 0.01, ^***^p < 0.001, ^****^p < 0.0001. The mice used for this analysis did not differ from the other animals in the given subgroup in terms of body weight, blood glucose and iron levels. Red line – the reference values of normoglycemic mice fed with high iron and standard diet (27).

3-month-old male BKS-Leprdb/db/JOrlRj (*db/db*) mice, obtained from the Janvier Labs (Saint Berthevin Cedex, France), were used in this study. The study was conducted according to the guidelines of the Declaration of Helsinki, and approved by the Landesdirektion Sachsen, Leipzig, the local authority for animal care (reg. no.: TVV65/15). Animals were divided into two groups of *db/db* mice, which were fed with different chows (Altromin, Lage, Germany) containing high iron (29 g/kg – standard diet modified with 3% carbonyl iron) or standard iron (0.178 g/kg) concentrations for 4 months. In our previous neurological work, we used *db/db* mice fed with different amounts of dietary non-heme iron for the first time (27). Iron is an essential element, but it is known that some forms of dietary iron could be toxic (30–32). To exclude the toxic effect of dietary carbonyl iron on the mice used in this study, we also tested *db/*+ mice as an additional control. All *db/*+ mice fed with high iron diet were normoglycemic and they could not be divided into subgroups of high and low glucose. Despite the high level of serum iron, they did not show any signs of metabolic and hematopoietic disorders and all investigated metabolic parameters in this control group were similar to that observed in the standard and low iron diet fed *db/*+ control groups (27). In the present study, we fed mice with the same high iron diet. Since the high iron fed *db/*+ mice have not shown any differences in blood glucose level, they have been excluded as an additional control from the present experiment. The experiment was repeated six times. Body weight, blood glucose concentration and HbA1c were measured as described (27). Lipid status and serum iron levels were performed by the Institute of Laboratory Medicine, Clinical Chemistry and Molecular Diagnostics, University of Leipzig. Serum insulin (Mouse Ultrasensitive Insulin ELISA, Alpco Diagnostics, Salem, NH), C-peptide (Mouse Ultrasensitive C-peptide ELISA, Alpco Diagnostics, Salem, NH) and adiponectin [Adiponectin (mouse) ELISA Kit, AdipoGen® LIFE SCIENCES, Liestal, Switzerland] concentrations were detected by ELISA according to the manufacturer's protocol. Serum concentrations of Chemokine ligand 2 (CCL2), Chemokine ligand 20 (CCL20), Cadherin-6 (Cdh6), Coxsackie virus and adenovirus receptor-like membrane protein (Clmp), Delta like non-canonical notch ligand 1 (DLK1), Erb-b2 receptor tyrosine kinase 4 (ErbB4), Follistatin like 3 (Fstl3) and Seizure related six homolog-like two (Sez16) were determined by mouse OLINK proteomics (Uppsala, Sweden). The first four experiments were performed as a part of previous neurological studies and parts of the values have already been used, but without dividing of the high iron group into two subgroups (body weight, blood glucose concentration, HbA1c, serum insulin, serum iron levels, see Figures 1A–G) (27).

### Immunostaining and Quantification of Pancreatic Islet ß-Cells

Pancreatic tissue was fixed with 4 % paraformaldehyde, embedded within paraffin and sliced into 7 μm sections. Immunostaining was performed as previously described (27). Three slides each containing three slices of five animals per group were analyzed. The mice used for this analysis did not differ from the other animals in the given subgroup in terms of body weight, blood glucose and iron levels. Slices were incubated with insulin antibody (1:2,000, Abcam) overnight at 4°C and secondary antibody Alexa 488 goat anti-rabbit (1:500) for 1 h at room temperature. Nuclei staining was performed with DAPI (1:10,000, Serva).

Immunofluorescence stained microscope slides were fully digitized at 20x magnification using a digital slide scanner (Pannoramic Scan II, 3D HISTECH Ltd., Budapest, Hungary) equipped with a quad band (DAPI/FITC/TRITC/Cy5) filter set. DAPI channel (blue) represents nuclei staining, FITC channel (green) represents insulin staining and TRITC channel (red) represents tissue autofluorescence. Images of the stained tissue slices were exported from data sets using CaseViewer (Version 2.3, 3D HISTECH Ltd., Budapest, Hungary) with pixel dimensions of 0.325 μm.

Image analysis was performed with Mathematica (Version 12.0, Wolfram Research, Inc., Champaign, IL, USA). Fluorescence images were imported and adjusted for brightness and contrast. Tissue area was segmented using Otsu's (cluster variance maximization) thresholding method (33), small disconnected tissue segments were removed and tissue artifacts like small cuts or fissures were corrected using a five pixel wide morphological closing filter, an operation which dilates the segmented tissue area by five pixels (enlarges the tissue area and closes gaps) and subsequently erodes the tissue by five pixels (reduces the tissue area but keeps gaps closed). Imported images were split into separate color channels. While tissue autofluorescence is negligible in the blue channel it can appear as bright as stained islets in the green channel. However, the autofluorescence is not limited to the green channel only as it also appears in the red channel with comparable intensity. Consequently, autofluorescence correction was performed by subtracting the red from the green channel. Supplemental figure shows the difference in segmented islets between original green channel and channel subtraction (green minus red). While detection results from the green channel (Supplementary Figures 1E,F) show many components or component appendages originating from unspecific staining (i.e. autofluorescence), only specifically stained and properly delimited islets remain in the detection results from channel subtraction (Supplementary Figures 1G,H). This comparison underlines the need for an autofluorescence correction method of the green channel and in this study we used fluorescence information already present in the red channel. The resulting images show minimal residue of autofluorescence along with brightly stained islets that can be easily detected. Pancreatic islets in the corrected green channel were also segmented using Otsu's thresholding method and subsequently enhanced by morphological closing since uncorrected islets may appear slightly porous due to the staining of individual islet cells. Segmented islets were visually examined and image masks for the removal (staining artifacts, tissue overlaps, blurry regions) or adding (poorly stained islets or islet regions) of structures were created by hand using GIMP (Version 2.10.2, The GIMP team, http://www.gimp.org) when necessary. These masks were subsequently imported in Mathematica and the respective islet segmentations were corrected. Based on the final segmentations, tissue and islet areas were calculated. Fluorescence intensity values of masked islets were accumulated both for high-intensity as well as low-intensity portions of all segmented islets. Finally, all values were normalized based on the tissue area of the respective images.

### RNA Extraction and cDNA Synthesis

Liver and visceral adipose tissues (vAT) were stored at −80°C. The mice used for this analysis did not differ from the other animals in the given subgroup in terms of body weight, blood glucose and iron levels. Tissues were transferred into 1,000 μl Trizol (Invitrogen), homogenized with Ultra Turax (IKA), centrifuged and the supernatant was collected. 200 μl Chloroform (Roth) was added, centrifuged and rinsed with ice cold isopropanol and ethanol. Pellet was solved in 30 μl RNase-free water. DNase digestion was carried out with TURBO DNA-free Kit (Invitrogen). RNA concentration was measured with a Qubit 3.0 Fluorometer (Invitrogen) and RNA BR Assay kit (Invitrogen). cDNA-Synthesis was performed with ProtoScript First Standard cDNA Synthesis Kit (BioLabs).

Quantitative RT-PCR with SYBR Green qPCR Master Mix (Thermo scientific) was performed using the CFX96 Real-Time System (Bio-Rad). mRNA expression of target genes was normalized to the expression of *IPO8, ATCB* and *B2M* mRNA. Values of the high iron fed subgroup 1 were compared with the values of subgroup 2. Primer sequences used are shown in the Supplementary Table.

### Hematoxylin-Eosin and Prussian Blue Staining

Liver and pancreatic paraffin slides were incubated with hematoxylin for 10 min for HE staining or with 2% potassium ferrocyanide and 2% hydrochloric acid for 30 min at room temperature for Prussian blue staining. Eosin or nuclear fast red were used for counterstaining. Stained microscope slides were fully digitized at 20x magnification using a digital slide scanner (Pannoramic Scan II, 3D HISTECH Ltd., Budapest, Hungary).

### Statistical Analysis

Data are presented as mean ± SD. Differences between the groups were validated by a two-tailed unpaired Student's *t*-test or one-way-ANOVA and the Tukey test for the adoption of a normal distribution or Kruskal-Wallis and Mann-Whitney for non-parametric test using GraphPad Prism Eight Values of *p* < 0.05 were considered as statistically significant.

## Results

### Phenotypic Characterization of High Iron Group and Development of Diabetic Alterations

Based on blood glucose concentrations after 4 months high iron diet, we divided this group of *db/db* mice into two subgroups: Subgroup 1 (Sub1) with blood glucose level lower than 13.5 mmol/l and subgroup 2 (Sub2) with blood glucose concentration equal to or higher than 13.5 mmol/l (Supplementary Figure 2). Notably, we did not observe such differences in the standard group (Supplementary Figure 2). Only two out of 42 animals showed a blood glucose level lower than 13.5 mmol/l (Supplementary Figure 2). At the start of the experiment there were no significant differences in blood glucose levels between the groups (Supplementary Figure 3). In Sub1, a significant decrease of blood glucose concentrations was observed during the experiment while in Sub2, blood glucose levels increased similarly to those observed in the standard group (Figures 1A–C). Surprisingly, body weight of Sub1 animals increased from 50 g (±3.5 g) to 59.4 (±5 g) while in Sub2 and in mice fed with standard iron diet, it decreased from 49.7 g (±3.5 g) to 47.2 (±8.5 g) and from 50 g (±4.3 g) to 46.6 g (±8.9 g), respectively (Figures 1B,D). In addition to blood glucose levels, the HbA1c content in Sub1 was significantly lower (6,3 % ± 2 %) as compared to Sub2 (11.97 % ± 2.6 %) and the *db/db* mouse standard group (9.8 % ± 2.8 %) (Figure 1E). Notably, serum insulin concentrations correlated with these findings and were increased in Sub1 (7.6 ng/ml ± 3.4 ng/ml) compared to Sub2 (1.4 ng/ml ± 1.2 ng/ml) as well as the standard group (2.9 ng/l ± 3.6 ng/l) (Figure 1F). Serum iron concentration of Sub1 was significantly higher (97.8 mmol/l ± 19.7 mmol/l) than in Sub2 (64.1 mmol/l ± 18.0 mmol/l). As expected, Sub2 showed a higher serum iron concentration compared to the *db/db* mice on standard diet (36.6 mmol/l ± 8.7 mmol/l) (Figure 1G).

In addition to the increased serum insulin concentrations, serum C-peptide levels in Sub1 were also higher than in Sub2 and standard group (Figure 1H). Furthermore, serum levels of adiponectin were comparable in subgroup 1, subgroup 2 and standard group (Figure 1I). Next, we analyzed the serum lipid status of high iron Sub1 and Sub2 and standard group. Parallel to increased body weight and serum insulin concentration, the serum total cholesterol level and the concentrations of its fractions (high-density-lipoprotein-cholesterol (LDL) and low-density-lipoprotein-cholesterol (HDL) were significantly higher in Sub1 compared to the Sub2 and standard group (Figure 1J). Notably, there were no differences in triglyceride and free fatty acid concentrations between both animal subgroups (data not shown).

Due to the lack of division into two subgroups of standard iron diet fed animals, which do not differ in blood glucose levels, we have only analyzed the differences between Sub1 and Sub2 of the high iron fed *db/db* mice in the further part of the present study.

The mouse OLINK proteomic panel analysis was carried out to examine the levels of serum circulating proteins, which play an important role in glucose and lipid metabolism as well as in inflammation. Among 91 tested proteins, only Eight were significantly different between both high iron subgroups (Table 1). We found higher levels of Ccl2, Cdh 6, Clmp, DLK1, Fstl3, and Sez6l2 in Sub1 compared to Sub2. Serum concentrations of Ccl20 and ErbB4 were significantly lower in Sub1 compared to Sub2 (Table 1).

**Table 1:** Serum protein levels.

	**High iron sub1 (*n* = 5)**	**High iron sub2 (*n* = 5)**
Ccl2	13.64 ± 0.18 ^**^	12.42 ± 0.31
Ccl20	8.66 ± 1.08 ^*^	10.74 ± 1.47
Cdh6	3.73 ± 0.2 ^***^	2.77 ± 0.36
Clmp	6.68 ± 0.21 ^*^	6.14± 0.34
Dlk1	3.82 ± 0.4 ^***^	2.49 ± 0.32
ErbB4	5.46 ± 0.15 ^*^	6.22 ± 0.27
Fstl3	6.30 ± 0.12 ^**^	5.81 ± 0.15
Sez6l2	5.24 ± 0.25 ^***^	4.60 ± 0.26

*Values represent means ± SD*

* *p < 0.05*,

** *p < 0.01*,

*** *p < 0.001 (t-test). The mice used for this analysis did not differ from the other animals in the given subgroup in terms of body weight, blood glucose and iron levels*.

### Quantification of Pancreatic Islet Area of ß-Cells

**Figure 2:**
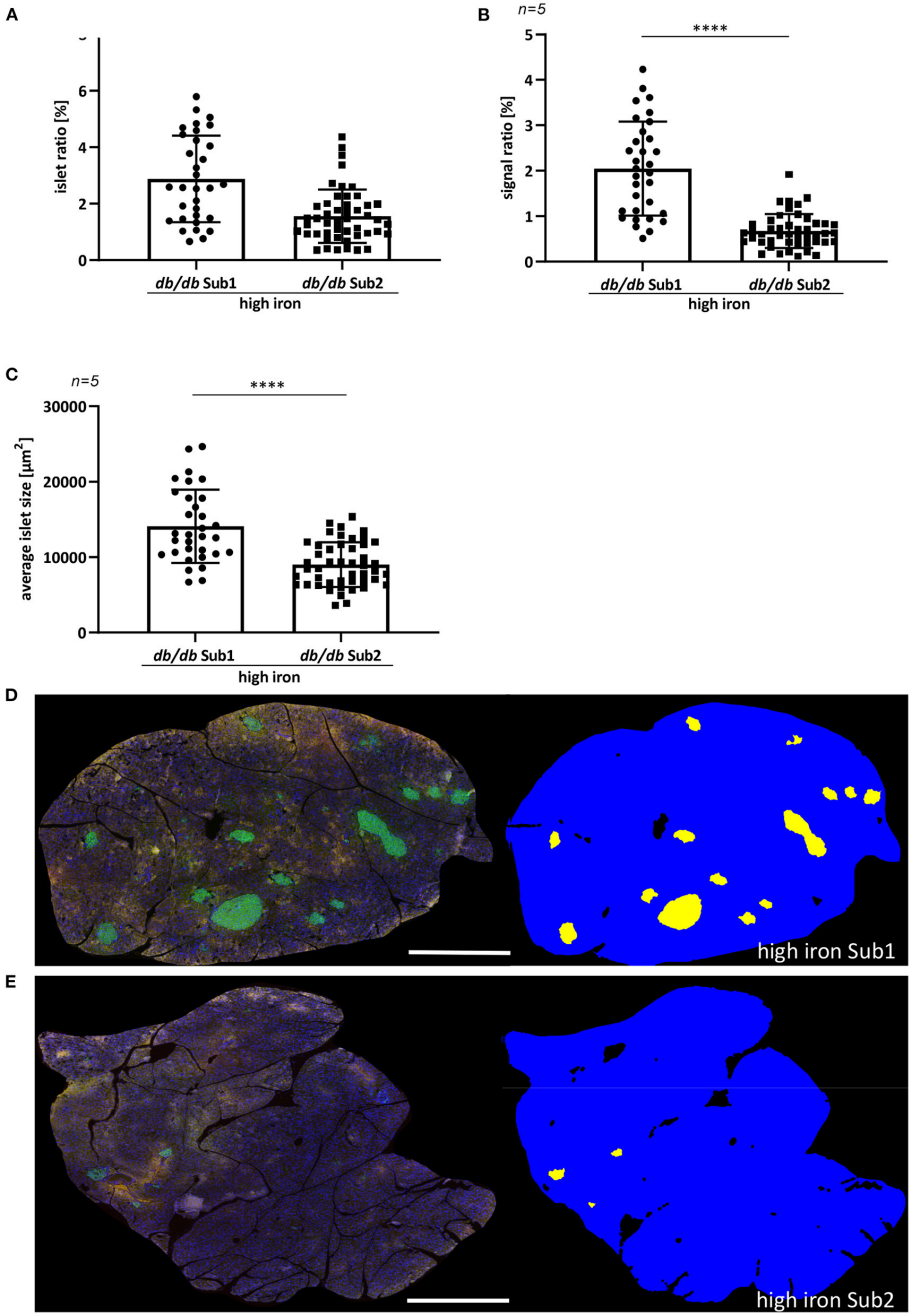
Quantification of pancreatic islet area of ß-cells. **(A)** Islet ratio (Mann-Whitney test). **(B)** Signal ratio of insulin (Mann-Whitney test). **(C)** Average islet size (*t*-test). **(D,E)** Immunofluorescence staining of insulin (green) and automatic detection of islet area in high iron Sub1 group **(D)** and Sub2 group **(E)**. Average islet area is larger than in subgroup 2 combined with a higher insulin signal ratio. *n* = 5 animals per group, 30–48 ß-cell islets per group were analyzed. Values represent means ± SD ****p < 0.0001. Bar represented 1,000 μm.

Previously, it has been shown that iron increases the apoptosis of pancreatic ß cells by inducing the production of ROS, which in turn promotes the development of diabetes mellitus (16–18). For this reason, we examined the pancreas morphology of *db/db* mice fed with high iron diet. An automatic quantification of pancreatic ß-cell islets was used to analyze differences in the signal ratio of insulin and average islet size (Figure 2). The analysis revealed a significant higher islet and signal ratio of insulin in pancreas of Sub1 compared to Sub2 (Figures 2A,B). Also, the average islet size was increased in Sub1 compared to Sub2 (Figure 2C).

### Markers of Inflammation and Macrophages in Liver and Visceral Adipose Tissue

**Figure 3:**
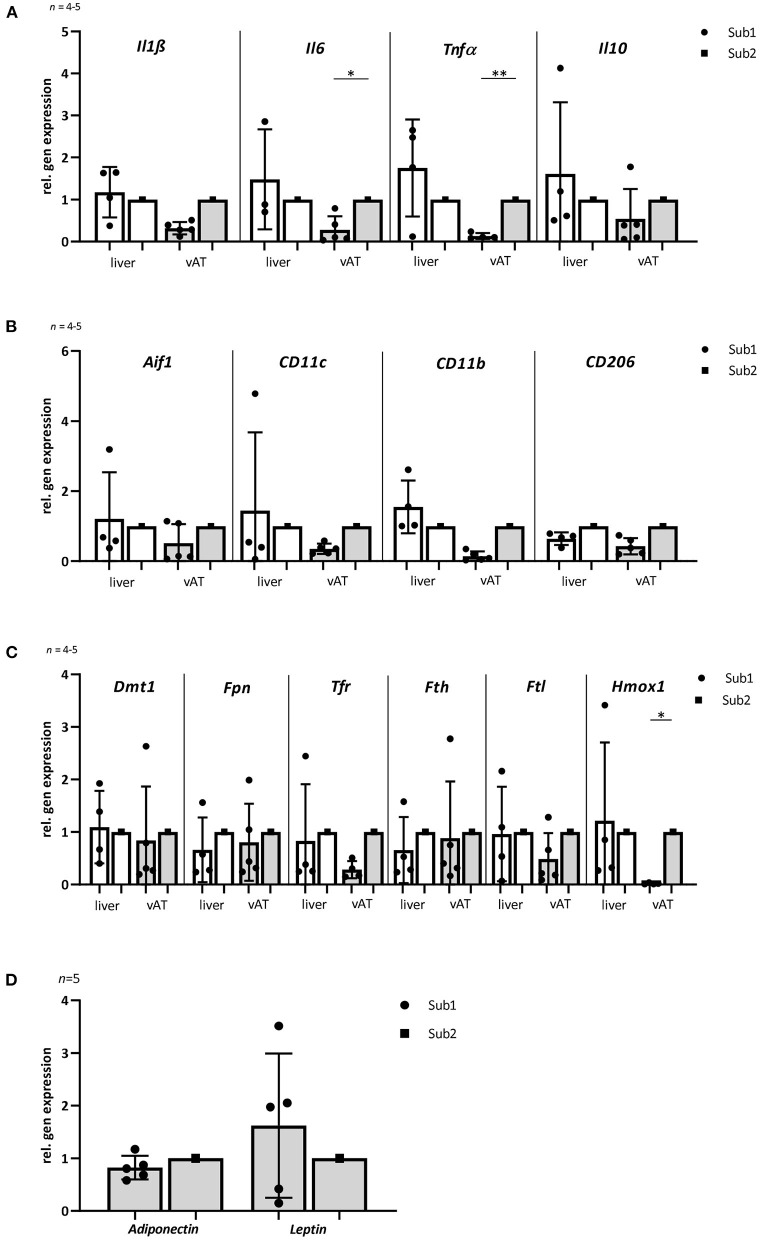
Gene expression of inflammatory, macrophage and iron homeostasis markers as well as adiponectin and leptin in liver and visceral adipose tissue of mice fed with high iron diet (*n* = 4–5). The mice used for this analysis did not differ from the other animals in the given subgroup in terms of body weight, blood glucose *a*nd iron levels. **(A)** Relative gene expression of inflammatory markers *Il1ß, Il6, Tnf*α, and *Il10*. The levels of inflammation markers in vAT are lower in subgroup 1 than in subgroup 2. No differences are observed in the liver. **(B)** Relative gene expression of macrophage markers: *Aif1, CD11c, CD11b*, and *CD206*. **(C)** Relative gene expression of iron metabolism markers: *Dmt1, Fpn, Tfr1, Fth, Ftl*, and *Hmox1*. Note that there are no differences in expression of most iron metabolism markers in the two *db/db* mouse subgroups in both tissues. Only *Hmox1* is significantly reduced in subgroup 1 in the vAT compared to subgroup 2. **(D)** Relative gene expression analyses of adiponectin and leptin in visceral adipose tissue. Subgroup 1 values were compared with subgroup 2 values, which were normalized to one. Values represent means ± SD *p < 0.05, **p < 0.01 (*t*-test, Mann-Whitney test).

The association between obesity, T2D and chronic tissue inflammation with infiltration of immune cells and increased expression of inflammatory markers has been shown in several previous studies (34, 35). Notably, macrophages, liver and visceral adipose tissue (vAT) play a significant role in systemic iron homeostasis (36). Therefore, we analyzed different inflammation and macrophage markers in liver and vAT after 4 months of high iron diet using RT-PCR. Relative gene expression analyses of pro-inflammatory markers Il1ß, Il6, and Tnfα showed no differences in liver between both subgroups. In vAT, a significant lower expression of Il6 and Tnfα was observed in Sub1 compared to Sub2. Whereas the expression of an anti-inflammatory marker Il10 in both tissues was similar in the subgroups (Figure 3A). Relative gene expression of macrophage marker allograft inflammatory factor 1 (Aif1) in liver as well as in vAT was comparable in both high iron subgroups. In both tissues, pro-inflammatory M1 macrophage markers (CD11c and CD11b) and anti-inflammatory M2 macrophage marker CD206 were equally expressed in both subgroups (Figure 3B). Relative gene expression analyses of adiponectin and leptin in the vAT revealed no differences in the two subgroups (Figure 3D).

### Markers of Iron Metabolism in Liver and vAT

**Figure 4:**
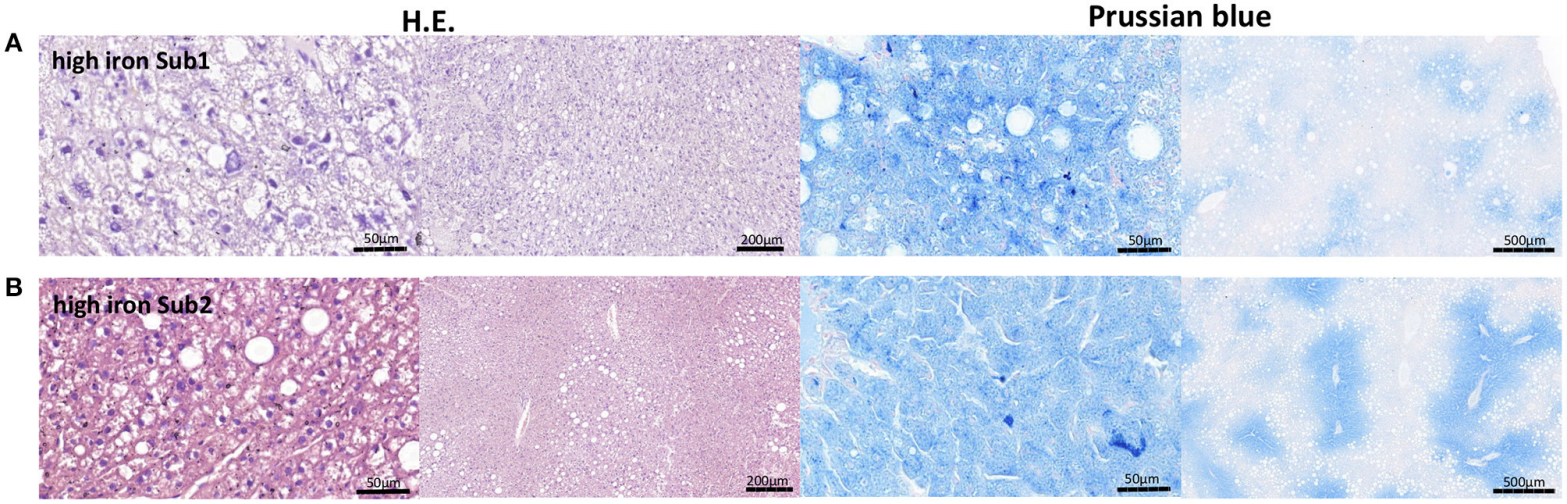
Representative H.E. and Prussian blue staining of the liver tissue. **(A)** High iron subgroup 1. **(B)** High iron subgroup 2. An accumulation of fat is in both subgroups recognizable. An iron deposition could be found in both high iron subgroups.

Liver is one of the main iron storage tissue, but iron can also be stored in vAT. Therefore, we investigated the main iron storage protein ferritin in both tissues using RT-PCR. Analysis of Fth and Ftl (ferritin heavy chain and ferritin light chain) gene expressions showed no differences in both subgroups. Next, we examined other iron metabolism markers such as divalent metal transporter 1 (DMT1), ferroportin (Fpn) and transferrin receptor 1 (Tfr1) in the liver as well as vAT. No differences in mRNA expression of the tested proteins were observed between two high iron subgroups. Only the heme oxygenase1 (Hmox1) level was significant reduced in the vAT of the Sub1 compared to the Sub2 (Figure 3C). Moreover, in the gut of both subgroups, we did not find any differences in mRNA expression of iron metabolism markers (data not shown). Then, we performed Prussian blue staining for visualization of iron in liver and pancreas. In the pancreas there were no detectable deposits of iron in all experimental groups (data not shown). Clusters of iron were visible in the liver tissue in Sub1 and Sub2. Iron deposits were found in sinusoids and hepatocytes with central alignment to the central vein. Analysis of HE-stained slices of all groups revealed an accumulation of fat in the liver (Figure 4).

## Discussion

### Correlation Between Glucose Metabolic Parameters, Pancreas Morphology and the Serum Iron Status

The principal finding of our study is that *db/db* mice fed for 4 months with high iron diet can be divided into two metabolic subgroups (Table 2). Our results of biochemical and morphological analyses clearly show a relationship between the improvement of glucose metabolic parameters and an increased volume of pancreatic islets with a simultaneous significant increase in insulin and C-peptide synthesis in Sub1. In context of these observations, the remarkably interesting finding of this study is increased circulating Sez6l2 protein levels in Sub1 mice. Sez6l2 occurs as a pancreatic islet-enriched cell surface protein and is expressed in endocrine progenitor islet cells during pancreatic development, although its role in islet cell division is still unknown (37). The increased level of this protein is associated with the increasing volume of the pancreatic islets in Sub1. The natural consequence of the above-mentioned changes was a reduction by half of the glucose level in this experimental group compared to the other mice. Notably, the highest level of serum iron has been also observed in Sub1, which indicates a possible direct or indirect protective effect of this essential but also potentially dangerous micronutrient on the ß-cells. Contrary to the present observations, Cooksey et al. have shown that dietary iron restriction or iron chelation protect from loss of β-cell function and diabetes in obese, leptin-deficient (*ob/ob*) mice (18). In their study, mice had hyperglycemia and were fed with high carbohydrate or high-fat diet with different iron content. Unfortunately, it could not be excluded here that the high carbohydrate or the high-fat diets were additional factors that might influence insulin and glucose metabolism and β-cell function of these leptin-deficient animals (18).

**Table 2:** Summary changes in biochemical and physiological parameters in subgroup 1 compared to subgroup 2 on high iron diet.

					**Pancreas**		**vAT**	
	**Blood glucose [mmol/l]**	**HbA1c [%]**	**Insulin conc. [ng/ml]**	**Lipids in serum [mmol/l]**	**Average islet size [μm^**2**^]**	**Signal ratio of insulin [%]**	**Inflammation**	**Hmox1**
Subgroup 1 vs. Subgroup 2	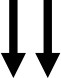	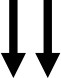	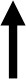	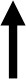	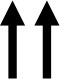	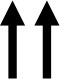	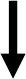	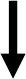

*Differences in biochemical and physiological parameters in serum, pancreas and vAT between the two subgroups. A single arrow - moderate differences; a double arrow - greater differences in subgroup 1 compared to subgroup 2*.

### Improved T2D Parameters and Body Weight Gain

An unexpected effect of the improvement of glucose metabolic parameters was a significant increase in body weight in Sub1 animals. It should be noted, however, that similar changes were observed in patients with too high doses of insulin (38). This may explain the increase in body weight in Sub1 mice, in whom insulin and C-peptide levels were significantly higher compared to the Sub2 animals. An interesting finding in the context of the above-mentioned results is the increased level of serum Clmp concentration observed in Sub1 mice. Clmp, also known as adipocyte adhesion molecule (ACAM), is a part of the epithelial tight junctions playing a role in cell-cell adhesion in white adipose tissue. Clmp is upregulated in adipocyte maturation in humans and rodents with obesity (39). Interestingly, the study of Murakami et al. with transgenic ACAM mice has shown a reduction in adipocyte mass with smaller adipocytes and a protection against obesity and diabetes (40). In contrast to these observations, the increased level of serum ACAM in the Sub1 mice correlated with a weight gain. The explanation of this phenomenon requires further investigations. Additionally, we found higher serum Fstl3 and DLK1 levels in Sub1 animals. Fstl3 shows glucose regulatory effects in rodents and humans (41, 42). Fstl3 is expressed and secreted in skeletal muscle. An overexpression of Fstl3 in mice fed with high-fat diet leads to fat gain and an improvement in insulin signaling (43–45). DLK1 (also called preadipocyte factor one) also plays a role in adipose tissue homeostasis and it is an inhibitor of adipogenesis by preventing adipocyte differentiation (46) as well as regulation of the whole-body glucose metabolism (47, 48). Surprisingly, we found lower serum concentration of ErbB4 in Sub1 combined with an improvement in several biochemical parameters of diabetes. ErbB4 is an important factor for cell proliferation, differentiation, migration and apoptosis (49). It is induced by different ligands such as neuroregulin. These ligands play an important role in glucose transport and lipogenesis (50, 51). ErbB4 deletion in mice fed with a moderate-fat diet leads to the development of metabolic syndrome with obesity, hyperglycemia, insulin resistance and hepatic steatosis (52). Further studies of adipocyte mass, size and physiology in accordance with the iron status are necessary to explain the exact role of the proteins mentioned here in the improvement of the diabetic status, the weight gain as well as the iron homeostasis in Sub1 and Sub2 animals.

The increased body weight of mice from Sub1 was accompanied by an increased level of cholesterol and its fractions, which is consistent with other studies published so far (53). Handa at el. examined male B6.BKS (D)-Leprdb/J diabetic mice fed with 2% carbonyl iron. They found hepatocellular ballooning in liver of diabetic iron-fed mice compared to control mice, which is a characteristic of human non-alcoholic steatohepatitis. Hepatic ballooning was combined with increased oxidative stress and inflammation as well as immune cell activation in liver of iron-fed mice. In accordance with our results, they also found higher serum cholesterol, LDL and HDL concentrations in serum of the high iron diet-fed *db/db* mice (54).

Interestingly, in another study, the opposite results regarding body weight in *db/db* mice fed with high iron diet have been demonstrated (32). However, in this experiment, the ferrous sulfate was used to enrich the diet with iron (32). Unlike this work, we used carbonyl iron. It is generally known that the ferrous sulfate is more toxic than carbonyl iron (30, 31). For this reason, it is impossible to exclude a toxic effect of ferrous sulfate in the applied dose, which would explain the loss of body weight in these high iron *db/db* animals (32).

### Body Weight Gain, Inflammatory Markers and Serum Iron Status

It is widely accepted that a major increase in adipose mass contributes to adipose tissue dysfunction. This dysfunction promotes metabolic disorders via a mild, chronic inflammation which is characterized by an increased expression of pro-inflammatory factors such as leptin, TNFα, IL6 or monocyte chemoattractant protein-1 (MCP-1 — also known as CCL2) (55). Interestingly, in the present study Tnfα and Il6 expression levels were the lowest in the vAT of Sub1 animals with higher body weight. Simultaneously, the serum iron concentrations were the highest in mice of this experimental group. Previously, we have shown that elevated serum iron levels are associated with lower Tnfα expression as well as decreased infiltration and activation of inflammatory cells in peripheral nerves, and consequently alleviation of the neuropathic symptoms in obese *ob/ob* and in obese and diabetic *db/db* mice (27, 28). Notably, the panel protein analysis revealed significant lower CCL20 serum concentrations in Sub1 mice. CCL20 stimulates the migration of dendritic cells as well as T and B cells (56) and it is upregulated by pathogenic bacteria and inflammatory cytokines such as Il1ß and TNFα (57–59). Moreover, it has been shown that an iron chelator deferoxamine (DFO) could induce the production of CCL20 in human intestinal epithelial cells (60). The observed decrease of CCL20 level in Sub1 mice correlates with lower Tnfα and Il6 expressions as well as higher serum iron concentration and it is consistent with previously published results. The obtained results clearly suggest a relationship between serum iron levels and vAT inflammation, glucose metabolism and body weight in both mouse subgroups fed with high iron diet.

Visceral fat plays a significant role in the regulation of iron homeostasis. Moreover, it is known as the main determinant of insulin resistance, and it is involved in the regulation of inflammation state through proinflammatory molecule production (36). For these reasons, we focused on changes in the visceral fat of *db/db* mice. Interestingly, Dongiovanni et al. have shown a decrease in visceral fat mass and no change in subcutaneous fat in mice fed a similar diet as in our study. In opposition to our results, the blood glucose levels were significantly higher in mice fed with high-iron diet (20). It should be noted here, however, that in the study by Dongiovanni et al., healthy, non-obese, wild-type mice (C57Bl/6) were used, which were also 6 weeks younger than our obese *db/db* animals at the beginning of the experiment. These observations confirm an important link between fat mass as well as iron and glucose metabolism, but a thorough explanation of the mechanisms of this interaction remains an open question that requires further studies including also subcutaneous fat.

### Iron Homeostasis Markers

All mice of similar genetic background fed with high iron diet kept in separate cages with free access to food have similar leptin levels but show differences in serum iron level concentrations between the two subgroups. To elucidate that in more detail, we analyzed markers of iron metabolism such as Dmt1, Fpn, Tfr, Fth and Ftl in liver, vAT as well as gut. We found no differences between the investigated animal groups. Based on these mRNA expression data we performed Prussian blue staining to detect iron in liver and pancreas. In pancreas there were no visible iron deposits in both subgroups. Accumulation of iron was found in livers of Sub1 and Sub2, but the amount was comparable. Taken together, observed differences in circulating iron levels in both subgroups cannot be explained by parameters of iron metabolism or iron storage capacity in the liver, vAT as well as in the gut.

Another important enzyme playing a crucial role in iron homeostasis is Hmox1. Handa et al. have previously shown a significant higher Hmox1 expression in the liver of *db/db* mice fed with high iron diet (54). Our analyses of Hmox1 expression show a significant reduction in vAT of Sub1 compared to Sub2 but no differences were observed in the liver. Hmox1 is induced by oxidative stressors such as ROS or pro-inflammatory cytokines (61, 62). The reports of Hmox1 in relation to disorders of glucose and lipid metabolism in connection with inflammation and iron homeostasis are unfortunately not conclusive. On the one hand, Hmox1 induction in parallel with an increase in serum adiponectin levels decreased pro-inflammatory markers such as TNFα, Il6 and Il1ß in obese mice (63). Moreover, Nicolai et al. (2009) showed that Hmox1 induction improved insulin sensitivity and decreased adiposity in Zucker diabetic rats (64). On the other hand, it has been shown that an increase in Hmox1 activity by heme results in decreased glucose uptake and increased markers of inflammation, oxidative stress and iron accumulation in human adipocytes (11, 24, 65). Here we found a decreased Hmox1 expression in vAT in combination with reduced expression of pro-inflammatory markers such as Tnfα and Il6 and CCL20 in Sub1. In contrast, a higher expression of Hmox1 in vAT was observed in Sub2 in combination with higher pro-inflammatory marker levels. Further metabolic and molecular studies are required to explain these differences in serum iron levels between both subgroups fed with high iron diet.

## Conclusion

In this study, we demonstrated the presence of two subpopulations of *db/db* mice within the high iron-fed group. These subpopulations differ in serum iron concentrations, pancreatic morphology, glucose, insulin, C-peptide levels, expressions of inflammatory markers, and body weight. These differences indicate a significant heterogeneity within this mouse strain especially in respect to glucose, lipid and iron metabolism and might be associated with impaired function of the pancreatic ß-cells. Moreover, we cannot exclude an impact of factors such as differences in food intake or absorption, metabolic rate, spontaneous activity, or individual insulin sensitivity. Further studies are required to explain these observations.

These findings may also explain the contradictory results of studies with *db/db* mice and show how important it is to define the metabolic profile of each tested animal. Based on observations presented in this study and the genetic diversity of patients with T2D, there may also be differences in response to the non-heme dietary iron. This should play an important role when devising an individual diet.

## Data Availability Statement

The original contributions presented in the study are included in the article/Supplementary Material, further inquiries can be directed to the corresponding author/s.

## Ethics Statement

The animal study was reviewed and approved by the local Ethics Committee of the state of Saxony (Landesdirektion Sachsen, Leipzig, approval nos. TVV 65/15).

## Author Contributions

SP, JK, and MN designed and planned the study. SP, KW, NK, PB, JK, and MN carried out the experimental work, biochemical, and statistical analysis. KW performed image acquisition and image processing. SP, IB, NK, MB, JK, and MN performed interpretation and discussion of results. SP, NK, JK, and MN drafted and revised the manuscript. All authors read and approved the final manuscript.

## Funding

This work was supported by grants of the Deutsche Diabetes Gesellschaft: DDG 934300-002 (MB and MN). The authors thank Andreas Horn and the staff from the animal facility for their excellent technical assistance. We acknowledge support from Leipzig University for Open Access Publishing.

## Conflict of Interest

The authors declare that the research was conducted in the absence of any commercial or financial relationships that could be construed as a potential conflict of interest.

## Publisher's Note

All claims expressed in this article are solely those of the authors and do not necessarily represent those of their affiliated organizations, or those of the publisher, the editors and the reviewers. Any product that may be evaluated in this article, or claim that may be made by its manufacturer, is not guaranteed or endorsed by the publisher.

## Abbreviations

ROS: reactive oxygen species
T2D: type 2 diabetes
Hmox1: hem oxygenase 1
TNFα: tumor necrosis factor α
Il: interleukine
ATMs: adipose tissue macrophages
Fth: ferritin heavy chain
Ftl: ferritin light chain
Fpn: ferroportin
Ccl2: chemokine ligand 2
Ccl20: chemokine ligand 20
Cdh 6: cadherin-6
Clmp: CXADR like membrane protein
DLK1: Delta like non-canonical Notch ligand 1
ErbB4: Erb-b2 receptor tyrosine kinase 4
Fslt3: Follistatin like 3
Sez6l2: Seizure related 6 homolog like 2
vAT: visceral adipose tissue
LDL: high-density-lipoprotein-cholesterol
HDL: low-density-lipoprotein-cholesterol
Aif1: allograft inflammatory factor 1
Dmt1: divalent metal transporter 1
Tfr1: transferrin receptor 1
ACAM: adipocyte adhesion molecule.

## References

[1] Galicia-Garcia U Benito-Vicente A Jebari S Larrea-Sebal A Siddiqi H Uribe KB. Pathophysiology of type 2 diabetes mellitus. Int J Mol Sci. (2020) 21:6275. 10.3390/ijms21176275 32872570 PMC7503727

[2] Roden M Shulman GI. The integrative biology of type 2 diabetes. Nature. (2019) 576:51–60. 10.1038/s41586-019-1797-8 31802013

[3] Khan MAB Hashim MJ King JK Govender RD Mustafa H. Al Kaabi J. Epidemiology of type 2 diabetes – global burden of disease and forecasted trends. J Epidemiol Glob Health. (2019) 10:107. 10.2991/jegh.k.191028.001 32175717 PMC7310804

[4] Hummel KP Dickie MM Coleman DL. Diabetes, a new mutafton in the mouse. Science. (1966) 153:1127–8. 10.1126/science.153.3740.1127 5918576

[5] Kaku K Province M Permutt MA. Genetic analysis of obesity-induced diabetes associated with a limited capacity to synthesize insulin in C57BL/KS mice: evicence for polygenic control. Diabetologia. (1989) 32:636-43. 10.1007/BF00274249 2676665

[6] Coleman DL Hummel KP. Symposium IV: diabetic syndrome in animals. Influence of genetic background on the expression of mutations at the diabetes locus in the mouse II Studies on background modifiers. Isr J Med Sci. (1975) 11:708−13. 1099054

[7] Naggert JK Mu J-L Frankel W Bailey DW Paigen B. Genomic analysis of the C57BL/Ks mouse strain. Mamm Genome. (1995) 6:131–3. 10.1007/BF00303258 7766997

[8] Mao HZ Roussos ET Péterfy M. Genetic analysis of the diabetes-prone C57BLKS/J mouse strain reveals genetic contribution from multiple strains. Biochim Biophys Acta. (2006) 1762:440–6. 10.1016/j.bbadis.2006.01.002 16481151

[9] Davis RC Schadt EE Cervino ACL Peterfy M Lusis AJ. Ultrafine mapping of snps from mouse strains C57BL/6J, DBA/2J, and C57BLKS/J for loci contributing to diabetes and atherosclerosis susceptibility. Diabetes. (2005) 54:1191–9. 10.2337/diabetes.54.4.1191 15793261

[10] Davis RC Castellani LW Hosseini M Ben-Zeev O Mao HZ Weinstein MM. Early hepatic insulin resistance precedes the onset of diabetes in obese C57BLKS-db/db mice. Diabetes. (2010) 59:1616–25. 10.2337/db09-0878 20393148 PMC2889760

[11] Bao W Rong Y Rong S Liu L. Dietary iron intake, body iron stores, and the risk of type 2 diabetes: a systematic review and meta-analysis. BMC Med. (2012) 10:119. 10.1186/1741-7015-10-119 23046549 PMC3520769

[12] Zhao Z Li S Liu G Yan F Ma X Huang Z. Body iron stores and heme-iron intake in relation to risk of type 2 diabetes: a systematic review and meta-analysis. PLoS ONE. (2012) 7:e41641. 10.1371/journal.pone.0041641 22848554 PMC3406072

[13] Miranda M Lawson H. Ironing out the details: untangling dietary iron and genetic background in diabetes. Nutrients. (2018) 10:1437. 10.3390/nu10101437 30301129 PMC6213605

[14] Pantopoulos K Porwal SK Tartakoff A Devireddy L. Mechanisms of mammalian iron homeostasis. Biochemistry. (2012) 51:5705–24. 10.1021/bi300752r 22703180 PMC3572738

[15] Salvador GA. Iron in neuronal function and dysfunction. BioFactors. (2010) 36:103–10. 10.1002/biof.80 20232345

[16] Andrews NC. Disorders of iron metabolism. N Engl J Med. (1999) 341:1986–95. 10.1056/NEJM199912233412607 10607817

[17] Rajpathak SN Crandall JP Wylie-Rosett J Kabat GC Rohan TE Hu FB. The role of iron in type 2 diabetes in humans. Biochim Biophys Acta. (2009) 1790:671–81. 10.1016/j.bbagen.2008.04.005 18501198

[18] Cooksey RC Jones D Gabrielsen S Huang J Simcox JA Luo B. Dietary iron restriction or iron chelation protects from diabetes and loss of β-cell function in the obese (*ob/ob lep* ^−/−^) mouse. Am J Physiol-Endocrinol Metab. (2010) 298:E1236–43. 10.1152/ajpendo.00022.2010 20354157 PMC2886527

[19] Fernandez-Cao JC Arija V Aranda N Bullo M Basora J Martínez-González MA. Heme iron intake and risk of new-onset diabetes in a Mediterranean population at high risk of cardiovascular disease: an observational cohort analysis. BMC Public Health. (2013) 13:1042. 10.1186/1471-2458-13-1042 24188615 PMC4228354

[20] Dongiovanni P Ruscica M Rametta R Recalcati S Steffani L Gatti S. Dietary iron overload induces visceral adipose tissue insulin resistance. Am J Pathol. (2013) 182:2254–63. 10.1016/j.ajpath.2013.02.019 23578384

[21] Gabrielsen JS Gao Y Simcox JA Huang J Thorup D Jones D. Adipocyte iron regulates adiponectin and insulin sensitivity. J Clin Invest. (2012) 122:3529–40. 10.1172/JCI44421 22996660 PMC3461897

[22] Jais A Einwallner E Sharif O Gossens K Lu TT-H Soyal SM. Heme oxygenase-1 drives metaflammation and insulin resistance in mouse and man. Cell. (2014) 158:25–40. 10.1016/j.cell.2014.04.043 24995976 PMC5749244

[23] Manuel Fernández-Real J Blasco G Puig J Moreno M Xifra G Sánchez-Gonzalez J. Adipose tissue R2^*^ signal is increased in subjects with obesity: a preliminary MRI study: adipose tissue and R2^*^. Obesity. (2016) 24:352–8. 10.1002/oby.21347 26813526

[24] Moreno-Navarrete JM Ortega F Rodríguez A Latorre J Becerril S Sabater-Masdeu M. HMOX1 as a marker of iron excess-induced adipose tissue dysfunction, affecting glucose uptake and respiratory capacity in human adipocytes. Diabetologia. (2017) 60:915–26. 10.1007/s00125-017-4228-0 28243792

[25] Moreno-Navarrete JM Ortega F Moreno M Ricart W Fernández-Real JM. Fine-tuned iron availability is essential to achieve optimal adipocyte differentiation and mitochondrial biogenesis. Diabetologia. (2014) 57:1957–67. 10.1007/s00125-014-3298-5 24973963

[26] Orr JS Kennedy A Anderson-Baucum EK Webb CD Fordahl SC Erikson KM. Obesity alters adipose tissue macrophage iron content and tissue iron distribution. Diabetes. (2014) 63:421–32. 10.2337/db13-0213 24130337 PMC3900546

[27] Paeschke S Baum P Toyka KV Blüher M Koj S Klöting N. The role of iron and nerve inflammation in diabetes mellitus type 2-induced peripheral neuropathy. Neuroscience. (2019) 406:496–509. 10.1016/j.neuroscience.2019.03.005 30867132

[28] Kosacka J Woidt K Toyka KV Paeschke S Klöting N Bechmann I. The role of dietary non-heme iron load and peripheral nerve inflammation in the development of peripheral neuropathy (PN) in obese non-diabetic leptin-deficient *ob/ob* mice. Neurol Res. (2019) 41:341–53. 10.1080/01616412.2018.1564191 30638160

[29] Baum P Kosacka J Estrela-Lopis I Woidt K Serke H Paeschke S. The role of nerve inflammation and exogenous iron load in experimental peripheral diabetic neuropathy (PDN). Metabolism. (2016) 65:391–405. 10.1016/j.metabol.2015.11.002 26975531

[30] Gordeuk V Brittenham G McLaren C Hughes M Keating L. Carbonyl iron therapy for iron deficiency anemia. Blood. (1986) 67:745–52. 10.1182/blood.V67.3.745.745 3947745

[31] Gordeuk VR Brittenham GM Hughes M Keating LJ Opplt JJ. High-dose carbonyl iron for iron deficiency anemia: a randomized double-blind trial. Am J Clin Nutr. (1987) 46:1029–34. 10.1093/ajcn/46.6.1029 3318377

[32] Ma W Feng Y Jia L Li S Li J Wang Z. Dietary iron modulates glucose and lipid homeostasis in diabetic mice. Biol Trace Elem Res. (2019) 189:194–200. 10.1007/s12011-018-1446-3 30027366

[33] Otsu N. A Threshold selection method from gray-level histograms. IEEE Trans Syst Man Cybern. (1979) 9:62–6. 10.1109/TSMC.1979.4310076

[34] Xu H Barnes GT Yang Q Tan G Yang D Chou CJ. Chronic inflammation in fat plays a crucial role in the development of obesity-related insulin resistance. J Clin Invest. (2003) 112:1821–30. 10.1172/JCI200319451 14679177 PMC296998

[35] Zhou J Zhou S. Inflammation: therapeutic targets for diabetic neuropathy. Mol Neurobiol. (2014) 49:536–46. 10.1007/s12035-013-8537-0 23990376

[36] Winn NC Volk KM. Hasty AH. Regulation of tissue iron homeostasis: the macrophage “ferrostat”. JCI Insight. (2020) 5:e132964. 10.1172/jci.insight.132964 31996481 PMC7098718

[37] Stützer I Selevsek N Esterházy D Schmidt A Aebersold R Stoffel M. Systematic proteomic analysis identifies β-site amyloid precursor protein cleaving enzyme 2 and 1 (BACE2 and BACE1) substrates in pancreatic β-cells. J Biol Chem. (2013) 288:10536–47. 10.1074/jbc.M112.444703 23430253 PMC3624435

[38] Home P Riddle M Cefalu WT Bailey CJ Bretzel RG del Prato S. Insulin therapy in people with type 2 diabetes: opportunities and challenges? Diabetes Care. (2014) 37:1499–508. 10.2337/dc13-2743 24855154 PMC5131884

[39] Raschperger E Engstrom U Pettersson RF Fuxe J. CLMP, a novel member of the CTX family and a new component of epithelial tight junctions. J Biol Chem. (2004) 279:796–804. 10.1074/jbc.M308249200 14573622

[40] Murakami K Eguchi J Hida K Nakatsuka A Katayama A Sakurai M. Antiobesity action of ACAM by modulating the dynamics of cell adhesion and actin polymerization in adipocytes. Diabetes. (2016) 65:1255–67. 10.2337/db15-1304 26956488

[41] Mukherjee A Sidis Y Mahan A Raher MJ Xia Y Rosen ED. FSTL3 deletion reveals roles for TGF-beta family ligands in glucose and fat homeostasis in adults. Proc Natl Acad Sci USA. (2007) 104:1348–53. 10.1073/pnas.0607966104 17229845 PMC1783105

[42] Perakakis N Kokkinos A Peradze N Tentolouris N Ghaly W Tsilingiris D. Follistatins in glucose regulation in healthy and obese individuals. Diabetes Obes Metab. (2019) 21:683–90. 10.1111/dom.13572 30393997 PMC6368471

[43] Brandt C Hansen RH Hansen JB Olsen CH Galle P Mandrup-Poulsen T. Over-expression of Follistatin-like 3 attenuates fat accumulation and improves insulin sensitivity in mice. Metabolism. (2015) 64:283–95. 10.1016/j.metabol.2014.10.007 25456456

[44] Henningsen J Rigbolt KTG Blagoev B Pedersen BK Kratchmarova I. Dynamics of the skeletal muscle secretome during myoblast differentiation. Mol Cell Proteomics. (2010) 9:2482–96. 10.1074/mcp.M110.002113 20631206 PMC2984231

[45] Laurentino GC Ugrinowitsch C Roschel H Aoki MS Soares AG Neves M. Strength training with blood flow restriction diminishes myostatin gene expression. Med Sci Sports Exerc. (2012) 44:406–12. 10.1249/MSS.0b013e318233b4bc 21900845

[46] Kilian TM Klöting N Blüher M Beck-Sickinger AG. Prenatal notch1 receptor blockade by protein delta homolog 1 (DLK1) modulates adipocyte size in vivo. Int J Obes. (2016) 40:698–705. 10.1038/ijo.2015.227 26499442

[47] Hudak CS Gulyaeva O Wang Y Park S-M Lee L Kang C. Pref-1 marks very early mesenchymal precursors required for adipose tissue development and expansion. Cell Rep. (2014) 8:678–87. 10.1016/j.celrep.2014.06.060 25088414 PMC4138044

[48] Wang Y Kim K-A Kim J-H Sul HS. Pref-1, a preadipocyte secreted factor that inhibits adipogenesis. J Nutr. (2006) 136:2953–6. 10.1093/jn/136.12.2953 17116701

[49] Stern D. ErbBs in mammary development. Exp Cell Res. (2003) 284:89–98. 10.1016/S0014-4827(02)00103-9 12648468

[50] López-Soldado I Niisuke K Veiga C Adrover A Manzano A Martínez-Redondo V. Neuregulin improves response to glucose tolerance test in control and diabetic rats. Am J Physiol-Endocrinol Metab. (2016) 310:E440–51. 10.1152/ajpendo.00226.2015 26714846

[51] Maeda S Imamura M Kurashige M Araki S Suzuki D Babazono T. Replication study for the association of 3 SNP loci identified in a genome-wide association study for diabetic nephropathy in European type 1 diabetes with diabetic nephropathy in Japanese patients with type 2 diabetes. Clin Exp Nephrol. (2013) 17:866–71. 10.1007/s10157-013-0797-5 23543049

[52] Zeng F Wang Y Kloepfer LA Wang S Harris RC. ErbB4 deletion predisposes to development of metabolic syndrome in mice. Am J Physiol-Endocrinol Metab. (2018) 315:E583–93. 10.1152/ajpendo.00166.2018 29944391 PMC6230712

[53] Gostynski M Gutzwiller F Kuulasmaa K Döring A Ferrario M Grafnetter D. Analysis of the relationship between total cholesterol, age, body mass index among males and females in the who monica project. Int J Obes. (2004) 28:1082–90. 10.1038/sj.ijo.0802714 15211364

[54] Handa P Morgan-Stevenson V Maliken BD Nelson JE Washington S Westerman M. Iron overload results in hepatic oxidative stress, immune cell activation, and hepatocellular ballooning injury, leading to nonalcoholic steatohepatitis in genetically obese mice. Am J Physiol-Gastrointest Liver Physiol. (2016) 310:G117–27. 10.1152/ajpgi.00246.2015 26564716

[55] Ellulu MS Patimah I. Khaza'ai H, Rahmat A, Abed Y. Obesity and inflammation: the linking mechanism and the complications. Arch Med Sci. (2017) 4:851–63. 10.5114/aoms.2016.58928 28721154 PMC5507106

[56] Schutyser E Struyf S Van Damme J. The CC chemokine CCL20 and its receptor CCR6. Cytokine Growth Factor Rev. (2003) 14:409–26. 10.1016/S1359-6101(03)00049-2 12948524

[57] Fujiie S Hieshima K Izawa D Nakayama T Fujisawa R Ohyanagi H. Proinflammatory cytokines induce liver and activation-regulated chemokine/macrophage inflammatory protein-3α/CCL20 in mucosal epithelial cells through NF-κB. Int Immunol. (2001) 13:1255–63. 10.1093/intimm/13.10.1255 11581170

[58] Izadpanah A Dwinell MB Eckmann L Varki NM Kagnoff MF. Regulated MIP-3α/CCL20 production by human intestinal epithelium: mechanism for modulating mucosal immunity. Am J Physiol-Gastrointest Liver Physiol. (2001) 280:G710–9. 10.1152/ajpgi.2001.280.4.G710 11254498

[59] Sierro F Dubois B Coste A Kaiserlian D Kraehenbuhl J-P Sirard J-C. Flagellin stimulation of intestinal epithelial cells triggers CCL20-mediated migration of dendritic cells. Proc Natl Acad Sci USA. (2001) 98:13722–7. 10.1073/pnas.241308598 11717433 PMC61108

[60] Lee H-J Choi S-C Choi E-Y Lee M-H Seo G-S Kim E-C. Iron chelator inducesMIP-3α/CCL20 in human intestinal epithelial cells: implication for triggering mucosal adaptive immunity. Exp Mol Med. (2005) 37:297–310. 10.1038/emm.2005.40 16155407

[61] Abraham NG Kappas A. Pharmacological and clinical aspects of heme oxygenase. Pharmacol Rev. (2008) 60:79–127. 10.1124/pr.107.07104 18323402

[62] Otterbein LE Choi AMK. Heme oxygenase: colors of defense against cellular stress. Am J Physiol-Lung Cell Mol Physiol. (2000) 279:L1029–37. 10.1152/ajplung.2000.279.6.L1029 11076792

[63] Li M Kim DH Tsenovoy PL Peterson SJ Rezzani R Rodella LF. Treatment of obese diabetic mice with a heme oxygenase inducer reduces visceral and subcutaneous adiposity, increases adiponectin levels, and improves insulin sensitivity and glucose tolerance. Diabetes. (2008) 57:1526–35. 10.2337/db07-1764 18375438

[64] Nicolai A Li M Kim DH Peterson SJ Vanella L Positano V. Heme oxygenase-1 induction remodels adipose tissue and improves insulin sensitivity in obesity-induced diabetic rats. Hypertension. (2009) 53:508–15. 10.1161/HYPERTENSIONAHA.108.124701 19171794 PMC2745551

[65] Arredondo M Jorquera D Carrasco E Albala C Hertrampf E. Microsatellite polymorphism in the heme oxygenase-1 gene promoter is associated with iron status in persons with type 2 diabetes mellitus. Am J Clin Nutr. (2007) 86:1347–53. 10.1093/ajcn/86.5.1347 17991645

