# Supplementary material for: Leptin Receptor-Deficient db/db Mice Show Significant Heterogeneity in Response to High Non-heme Iron Diet
Source: Front Nutr. 2021 Sep 27;8:741249. doi: 10.3389/fnut.2021.741249 (PMC8503537; doi:10.3389/fnut.2021.741249)
Supplement: Supplementary file 1 [file Data_Sheet_1.docx]

**Leptin receptor-deficient *db/db* mice show significant heterogeneity in response to high non-heme iron diet**

Sabine Paeschke^1^, Karsten Winter^1^, Ingo Bechmann^1^, Nora Klöting^2^, Matthias Blüher^2,3^, Petra Baum^4^, Joanna Kosacka^3,5†^ and Marcin Nowicki^1†^

^1^ Institute of Anatomy, University of Leipzig, Germany. ^2^ Helmholtz Institute for Metabolic, Obesity and Vascular Research (HI-MAG) of the Helmholtz Zentrum München at the University of Leipzig, Leipzig, Germany. ^3^ Department of Medicine, University of Leipzig, Leipzig, Germany. ^4^ Department of Neurology, University of Leipzig, Leipzig, Germany. ^5^ Department of Visceral, Transplant, Thoracic and Vascular Surgery, Applied Molecular Hepatology Lab, University of Leipzig Medical Center, Leipzig, Germany. ^†^ These authors have contributed equally to this work and share senior authorship

Correspondence: Marcin Nowicki

Institute of Anatomy

University of Leipzig

Liebigstr. 13

04103 Leipzig

Germany

Marcin.Nowicki@medizin.uni-leipzig.de

**Supplemental Table**: Primer sequences

|  | forward | reverse |
| --- | --- | --- |
| Il-1ß | TGCCACCTTTTGACAGTGATG | TGATGTGCTGCTGCGAGATT |
| Il-10 | GGCGCTGTCATCGATTTCTC | ATGGCCTTGTAGACACCTTGG |
| Il-6 | AGTTCCTCTCTGCAAGAGACTTC | AAGTAGGGAAGGCCGTGGTT |
| TnFα | GTCCCCAAAGGGATGAGAAGT | GCTCCTCCACTTGGTGGTTT |
| CD11c | GCAGACACTGAGTGATGCCA | TCGGAGGTCACCTAGTTGGG |
| CD11b | CATCCCCCTGCAAGTACCTC | GGGGGACAGTAGAAACAGCC |
| CD206 | GGCTGATTACGAGCAGTGGA | CATCACTCCAGGTGAACCCC |
| Aif1 | ATCAACAAGCAATTCCTCGATGA | CAGCATTCGCTTCAAGGACATA |
| Dmt1 | GCAGTGGTTAGCGTGGCTTATT | AGACAGACCCAATGCAATCAAA |
| Fpn | CCCTGCTCTGGCTGTAAAAG | GGTGGGCTCTTGTTCACATT |
| Tfr | GTTTTTGTGAGGATGCAGACTATCC | GCTGAGGAACTTTCTGAGTCAATG |
| Fth | TGATGAAGCTGCAGAACCAG | GTGCACACTCCATTGCATTC |
| Ftl | AATGGGGTAAAACCCAGGAG | AGATCCAAGAGGGCCTGATT |
| Hmox1 | TGCTCGAATGAACACTCTGG | AAGGCGGTCTTAGCCTC |
| Adiponectin | TGACGACACCAAAAGGGCTC | CACAAGTTCCCTTGGGTGGA |
| Leptin | TGACACCAAAACCCTCATCA | TGAAGCCCAGGAATGAAGTC |
| Ipo8 | ACAAGCTCTGCTGACTGTGC | CAGTGTCCTTCGGTGCTCTG |
| B2M | CTCGGTGACCCTGGTCTTTC | TTGAGGGGTTTTCTGGATAGCA |
| Atcb | CTGTCGAGTCGCGTCCA | TCATCCATGGCGAACTGGTG |

**Supplemental Figure** **1**


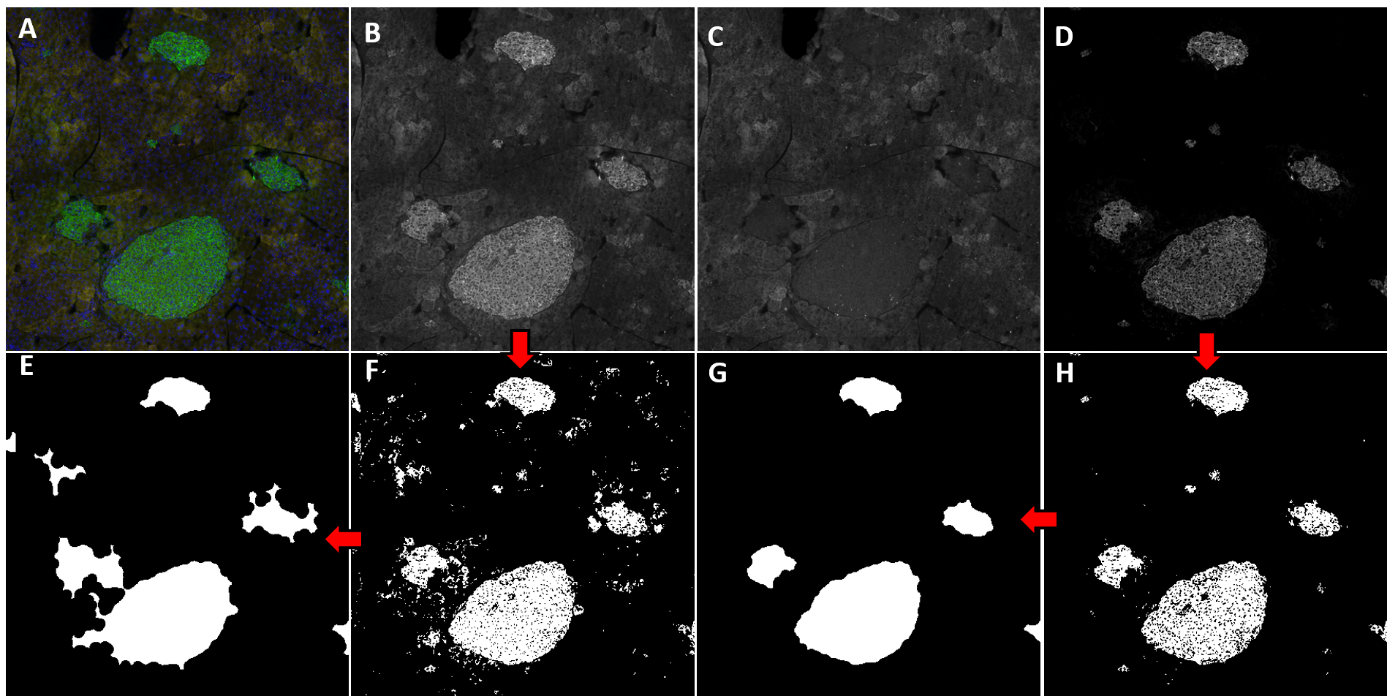


**Comparison between detection results of the green channel and channel subtraction** (green minus red). (A) Section of the original image. (B) Green channel. (C) Red channel. (D) Channel subtraction (green minus red channel). (E) Corrected segmentation of the green channel. (F) Raw segmentation of the green channel. (G) Corrected segmentation of channel subtraction. (H) raw segmentation of channel subtraction. Red arrows: Processing steps from grayscale images to corrected segmentation.

**Supplemental Figure** **2**

**Blood glucose concentration in mice fed with high iron (*n*=57) and standard diet (*n*=42) after 4 months.** A separation into two subgroups of the high iron group is recognisable. Separation line was manually set at 13.5 mmol/l. Experiments were repeated six times.

**Supplemental Figure 3**

**Blood glucose concentration in mice fed with high iron (*n*=57) and standard diet (*n*=42) before starting the diet at 12 weeks of age.** At the start of the experiment there were no significant differences in blood glucose levels between the groups. p values are above the graphs (ANOVA).
